# The Gut Microbiota Composition of *Cnaphalocrocis medinalis* and Their Predicted Contribution to Larval Nutrition

**DOI:** 10.3389/fmicb.2022.909863

**Published:** 2022-05-20

**Authors:** Chuanming Li, Guangjie Han, Jun Sun, Lixin Huang, Yurong Lu, Yang Xia, Qin Liu, Jian Xu

**Affiliations:** ^1^Department of Applied Microbiology, Jiangsu Lixiahe Institute of Agricultural Sciences, Yangzhou, China; ^2^National Experimental Station of Yangzhou for Agricultural Microbiology, Yangzhou, China; ^3^Yangzhou Luyuan Bio-Chemical Co., Ltd., Yangzhou, China

**Keywords:** *Cnaphalocrocis medinalis*, gut microbiota, metagenomic, nutrition, function prediction

## Abstract

Intestinal bacterial flora plays an important role in the nutrition, physiology, and behavior of herbivorous insects. The composition of gut microbiota may also be affected by the food consumed. *Cnaphalocrocis medinalis* is an oligophagous pest, feeds on rice leaves almost exclusively and causes serious damage to rice in Asian countries. Using antibiotic treatment and metagenome sequencing, we investigated the influence of the food sources (rice and maize seedlings) on the structure and functions of intestinal bacteria of *C. medinalis*. Firstly, food utilization indices, relative growth rate (*RGR*), relative consumption rate (*RCR*), efficiency of conversion of ingested food (*ECI*), and efficiency of conversion of digested food (*ECD*), were all significantly adversely affected in the antibiotic treatment eliminating gut bacteria, showing that the microbiota loading in the gut were essential for the larva growth and development of *C. medinalis*. Further, metagenome sequencing revealed that different diets caused a variation in gut microbiota composition of *C. medinalis*, indicating that the gut microbiota were in part driven by the diet provided. However, the larvae of *C. medinalis* hosted a core microbial community in the gut, which was independent from the diets changing. The dominant bacteria in the two feeding groups were highly consistent in the gut of *C. medinalis* larvae, with the gut bacterial community dominated by Firmicutes at the phylum level, *Enterococcus* at the genus level, *Enterococcus* sp. FDAARGOS-375, *E. casseliflavus*, *E. gallinarum*, and *E.* sp. CR-Ec1 accounted for more than 96% of the gut microbiota. Functional prediction analysis demonstrated that gut bacteria encoded a series of metabolism-related enzymes involved in carbohydrate metabolism and amino acid synthesis. Carbohydrate metabolism was the most enriched function in both groups and was more abundant in rice feeding group than in maize feeding group. The core dominant *Enterococcus* species possessed complete pathways of 14 carbohydrates metabolism, 11 amino acids biosynthesis, and two vitamins synthesize, implied to contribute an essential role to the nutrition intake and development of *C. medinalis*. Finally, the study may provide an in-depth analysis of the symbiont-host co-adaptation and new insights into the management of *C. medinalis*.

## Introduction

A large number of microorganisms coexist in the intestinal tract of insects. The symbiosis between microorganisms and their insect hosts is ubiquitous (Gupta and Nair, 2020). This relatively stable symbiotic relationship is critical to the entire life cycle of insects. Evidence showed that the gut microbiome had an important influence on insect nutrition intake, growth, reproduction, and immunity (Douglas, 2015; Strand, 2018; Jing et al., 2020; Kucuk, 2020).

The composition and diversity of insect intestinal microbes are affected by many factors, including environmental factors, the structure and physicochemical properties of insect intestines, and the food they consume (Voirol et al., 2018). Food is a major exogenous factor that directly influences the composition and metabolic capabilities of insect gut microbials (Majumder et al., 2020). The influence of diet on intestinal microbes is significantly stronger than that of the geographical environment (Priya et al., 2012). The gut bacterial populations of *Spodoptera frugiperda* increased in corn-fed larvae, while sharply decreased in the larvae that maintained on wheat germ diet (Mason et al., 2020). Recent research showed that it was difficult to detect any resident, food-independent intestinal bacteria in Lepidoptera (Staudacher et al., 2016; Hammer et al., 2017; Voirol et al., 2018). It seemed that only temporary and non-resident bacteria were allowed to host in the larval gut of lepidopterans. The gut microbial communities of lepidopterans should be similar to the microbiota of the food ingested. But other investigations showed lepidopterans hosted a core microbial community in their guts, which was distinctive from that obtained from the food source (Mason et al., 2020; Mazumdar et al., 2021). The importance of microorganisms in larval lepidopterans is presently debatable. Non-conclusive clustering patterns have been discovered, owing to the small number of current studies (Colman et al., 2012; Voirol et al., 2018).

Gut microbes of insects play an important role in the process of host adaptation to plants (Hammer and Bowers, 2015; Acevedo et al., 2017). *Plutella xylostella* gut microbes were found to participate in plant cell wall destruction and collaborate with their host to manufacture missing amino acids, which might affect the growth and development of host insects (Xia et al., 2017). *Enterobacter cloacae* could increase pupal weights and male fitness of *P. xylostella* under aseptic rearing conditions (Somerville et al., 2019). Feeding on an antibiotic-supplemented diet to reduce bacterial loads significantly decreased the nutritional indices relative growth rate (*RGR*) and relative consumption rate (*RCR*) of the *Spodoptera litura* larvae (Xia et al., 2020). However, another study found that using antibiotics did not affect the survival, growth, or development of *Danaus chrysippus* and *Ariadne merione* larvae (Phalnikar et al., 2019). It also suggests that research into the existence of true symbionts associated with Lepidoptera is still limited.

The rice leaf folder, *Cnaphalocrocis medinalis* Güenée (Crambidae, Lepidoptera), is one of the most damaging leaf-feeding pests in rice-growing regions throughout the southeast and northeast Asia (Khan et al., 1988; Savary et al., 2000; Park et al., 2010; Longkumer and Misra, 2020). Though it can complete a life cycle on maize seedlings and some other graminaceous plants in the laboratory (Liao et al., 2012), the larvae of *C. medinalis* are almost only exclusively in rice (Khan et al., 1996; Liu et al., 2012; Zhang et al., 2019). Using traditional isolation and culture methods, Yang (2012) obtained 25 species of 15 genus microbiota from *C. medinalis*, most of them belong to Bacilli and γ-Proteobacteria. Further analysis identified a total of 395 species across the full life cycle of *C. medinalis* by Illumina MiSeq technology, the common dominant bacteria were Proteobacteria and Actinobacteria based on phyla, and Actinobacteria, Acidobacteria, α-Proteobacteria, β-Proteobacteria, and γ-Proteobacteria at the class level (Yang et al., 2020). But it was still unclear whether there were dominant resident bacteria in the gut of *C. medinalis*. Under the present study, the composition and potential functions of gut microbes of *C. medinalis* feeding on rice and maize seedling were investigated using metagenomic sequencing. The effects of gut bacteria on food utilization of larvae were also investigated using axenic rearing methods. Our objectives were to: (1) characterize and compare the gut bacterial diversity of *C. medinalis* between different plants to further clarify the presence of any resident gut microbiome and (2) reveal the predicted functions of gut bacteria and their effects on feeding and growth that could provide an in-depth analysis of the symbiont-host co-adaptation and new insights into the management of *C. medinalis*.

## Materials and Methods

### Insect and Plant Sources

*Cnaphalocrocis medinalis* adults were collected from the rice paddies in the suburb of Yangzhou City, China (32°24′N, 119°26′E). According to the methods described previously (Shono and Hirano, 1989), the adults were given a 10% honey solution, and the larvae were reared with rice and maize seedlings and kept for more than 10 generations. Yangfujing 8 is a japonica rice variety that has been identified as a *C. medinalis* susceptible variety (Wang et al., 2008). Maize variety is Suyu 24, which is also susceptible to *C. medinalis*, as shown in our previous research (Li et al., 2019). Both adults and larvae were maintained at (25 ± 1)°C, 70% relative humidity, and a 16 h L:8 h D photocycle.

### Antibiotic Application

A mixture solution of 0.5 mg/ml tetracycline, ampicillin, clarithromycin, and erythromycin in sterile water was first prepared. Leaves of maize seedlings were cut into 5 cm pieces and dipped in the mixture of antibiotics for 30 s, dried in the air, and then placed into plastic Petri dishes. The fourth-instar *C. medinalis* larvae individual was reared on the treated maize seedling leaves (renewed at 24 h) for 48 h. A control group was prepared similarly with the leaves dipped in sterile water only. The effect of antibiotics on gut microbes was validated by incubating on LB basal medium. Ten healthy larvae were randomly selected and starved for 6 h before dissection, the larvae were surface-sterilized with 75% ethanol for 30 s and rinsed three times in sterile water, then the larvae were dissected under aseptic condition, the midgut contents were homogenized with 2 ml sterile water and diluted 10,000 times to spread plate. About 100 μl diluent was used to inoculate LB basal medium (yeast extract 5.0 g/L, peptone 10.0 g/L, NaCl 10.0 g/L, agar powder 15.0 g/L, and pH 7.0). The cultured gut bacteria were checked after 48 h in an incubator at 37°C.

### Food Utilization Indices Analysis

Ten healthy fourth-instar larvae of *C. medinalis* reared with maize seedling were selected and starved for 6 h, and then individually weighed before and after dried at 55°C to calculate the water content of fresh larvae. Based on the water content, another 30 healthy fourth-instar larvae were taken to be weighed individually after starvation for 6 h to estimate the dry weight of larvae before the experiment (*C*). Individual larvae were reared on antibiotics treated and control maize seedling leaves (renewed at 24 h) for 48 h and then dried at 55°C after being starved for 6 h to complete defecation. The dry weight of larvae after experiment (*D*), dry weight of feces (*E*), and dry weight of leaves before (*A*) and after (*B*) experiment were recorded. The nutrition indices included *RGR*, *RCR*, approximate digestibility (*AD*), efficiency of conversion of ingested food (*ECI*), and efficiency of conversion of digested food (*ECD*) were calculated by the following formula as described before (Wang, 1997):


$$RGR=\frac{D-C}{\frac{C+D}{2}\times T};RCR=\frac{A-B}{\frac{C+D}{2}\times T};AD=\frac{A-B-E}{A-B}\times 100;$$

$$ECI=\frac{D-C}{A-B}\times 100;ECD=\frac{D-C}{A-B-E}\times 100$$

### Gut Dissection and DNA Extraction

A total of 30 healthy individuals of fourth-instar *C. medinalis* larvae feeding on rice and maize seedling were selected, respectively, for each replicate, and three replicates were collected for each treatment. All the larval samples were first starved for 4 h before dissection, and then surface was sterilized in 75% ethanol for 30 s and rinsed three times in sterile water. The entire gut tissue was dissected and placed in 2 ml sterile tubes for DNA extraction under aseptic conditions. QIAamp DNA Stool Mini Kit (Qiagen, United States) was used for total metagenomic DNA extraction. The prepared intestinal sample was frozen in liquid nitrogen, fully ground, poured into 2 ml sterile tubes, and added 180 μl buffer ATL, and the following operation steps were carried out according to the kit instructions. DNA integrity was observed by electrophoresis in agarose gel. Microeco Tech Co., Ltd. in Shenzhen, China, was entrusted with sequencing the total DNA.

### Metagenomic Sequencing and Data Preprocessing

DNA library was constructed following the manufacturer’s instructions (Illumina, San Diego, CA, United States). We selected the paired-end (PE) library with a 350 bp insert size. All the samples were sequenced by the PE150 (2 × 150) protocol on the Illumina Novaseq 6000.

Because the raw data obtained by the Illumina sequencing platform contain a significant amount of low-quality data, it must be preprocessed to ensure the accuracy of subsequent analysis. Trimmomatic was used to remove the adapter (parameters: ILLUMINACLIP: adapters_path: 2: 30: 10; Bolger et al., 2014). We scanned the sequence (4 bp sliding window) and removed the subsequent sequence based on the average quality score is lower than 20 (99% accurate, parameters: SLIDINGWINDOW: 4: 20), or the reads <50 bp (parameters: MINLEN: 50). Filter of the host sequencing (parameters: very-sensitive) was conducted with Bowtie2 (Langmead and Salzberg, 2012), and the closely related species *Chilo suppressalis* (Walker) was chosen as host genome database. Finally, FastQC was used to test the logic and impact of quality control to obtain clean data.

### Metagenomic Assembly

We first performed assembly analysis using the MEGAHIT assembly software to obtain sample contigs (parameters:—k-list 21,29,39,59,79,99,119,141—min-contig-len 500; Li et al., 2015). Then, the clean data were mapped to the assembled contigs using Bowtie2 to get the unused PE reads (parameters:—end-to-end,—sensitive), put the unused PE reads together and hybrid assembled in MEGAHIT. The contigs with the length < 500 bp were filtered out. After that, we got hybrid assembly contigs of all samples.

### Gene Prediction and Annotation

The Prodigal software was used to predict gene sequences from all samples’ assembled contigs (Hyatt et al., 2010). Using Cd-hit to remove all redundant genes (Li and Godzik, 2006), the clean data were mapped to non-redundant genes to calculate the RPM (reads per million) of the non-redundant genes using Salmon software (parameters:—validate Mappings–meta; Patro et al., 2017). The non-redundant genes are translated into a protein sequence using Emboss software’s “Transeq” command, which may then be used in future alignments and annotations.

The annotations and classifications of all sequences were performed in Kraken2 (parameters:—confidence 0.2). Kraken2 scans every *k*-mers in the sequences and matches them to the LCA of genomes that contain the given k-mers (Wood and Salzberg, 2014). The result of Kraken2 was classified and re-evaluated based on Bayesian estimation in Bracken software to estimate the species level of metagenomic samples (Cotillard et al., 2013; Buttigieg and Ramette, 2014; Lu et al., 2016).

### Gene Function Annotation

The non-redundant protein sequences were BLASTed against the functional databases EggNOG (parameters:—seed_ortholog_evalue 0.00001) to obtain the annotation information, including KEGG, GO, and COG using eggNOG-Mapper software, carbohydrates biodegradation, amino acid, and vitamin synthesis functions were predicted *via* KEGG pathways (Huerta-Cepas et al., 2017). The annotation information for carbohydrate-active enzymes (CAZymes) was obtained from the CAZy database using DIAMOND software (Buchfink et al., 2015).

### Statistical Analysis

The statistical significance of the food utilization indices data between antibiotic treatment and control was analyzed with the Student’s *t*-test for unpaired comparisons, and a value of *p* of less than 0.05 was considered to be statistically significant. T-test analyses were conducted by GraphPad Prism (8.0.2). The interactive visualization of identified taxonomic data was depicted using the Krona program (Ondov et al., 2011). Principal component analysis (PCA) was done according to genera proportion at the best taxonomic level by R v.3.4.1.^1^ The comparison of microbial variations was analyzed by Kruskal Wilcox tests across taxonomic levels using R 3.4.1 and linear discriminant analysis effect size (LEfSe, LDA > 2; https://huttenhower.sph.harvard.edu/galaxy/; Huan et al., 2020). To explore functional differences, Statistical Analysis of Metagenomic Profiles (STAMP) was performed to compare the relative abundances of KEGG, CAZy, and eggNOG pathway categories (Parks et al., 2014).

## Results

### Effect of Gut Microbiota on Food Utilization Indices of *Cnaphalocrocis medinalis*

**Figure 1:**
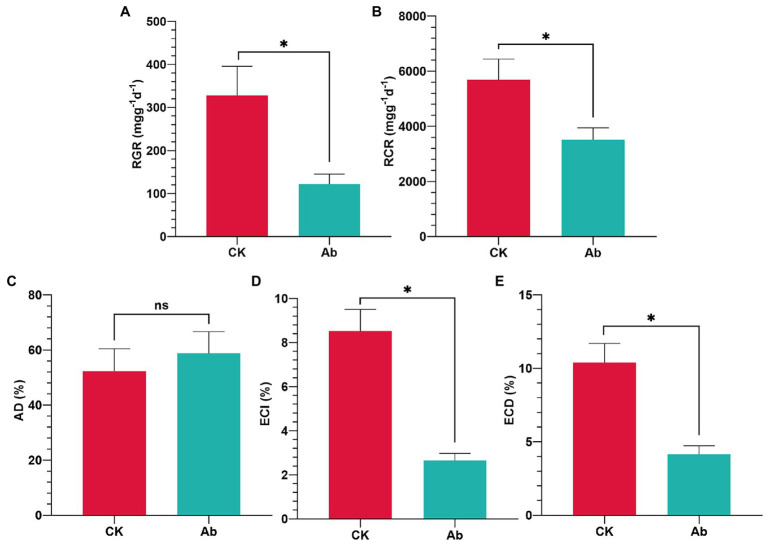
Effect of antibiotics on food utilization indices of *Cnaphalocrocis medinalis*. **(A)** Relative growth rate of *C. medinalis*; **(B)** Relative consumption rate of *C. medinalis*; **(C)** Approximate digestibility of *C. medinalis*; **(D)** Efficiency of conversion of ingested food of *C. medinalis*; and **(E)** Efficiency of conversion of digested food of *C. medinalis*. Ab, Antibiotic treatment; and CK, Control. Values are mean ± SE. ^*^Indicates significant difference at *p* < 0.05; *ns* indicates no significant difference (*p* ≥ 0.05).

The results of LB medium culture revealed that using antibiotics in combination effectively removed culturable bacteria from the intestinal tract (Supplementary Figure 1). Antibiotic treatment had significant effects on the food utilization indices of *C. medinalis* larvae (Figure 1). Compared with the control group, eliminating components of the intestinal flora significantly decreased the RGR (−62.96%), RCR (−38.22%), ECI (−68.90%), and ECD (−60.08%; *p* < 0.05; Figures 1A,B,D,E). while the AD of *C. medinalis* had no significant change (*p* ≥ 0.05; Figure 1C).

### Metagenomic Sequencing Summary

The metagenomic sequencing data yielded a total of 6.840 ± 0.08 and 7.067 ± 0.34 Gb raw bases of gut microbe genes of *C. medinalis* feeding with rice and maize seedlings, respectively. There was no significant difference between the two groups of raw bases (*p* = 0.372, *t* = 1.115, *df* = 2.192). After eliminating low-quality sequences, an average of 92.61 and 92.64% cleaned reads were recovered (Supplementary Table 1). Assembly analysis revealed a total length of 2955.93 Mb contigs, and then reduced to 1205.23 Mb after filtering by length 1,000 bp. The length of the largest contig was 328,345 bp, and the percentage of GC content was 38.52% (Supplementary Table 2). A total of 1,048,576 ORFs were found according to the contigs. The ORF length was distributed between 60 and 12,972 bp, and the average length was 204.71 bp. The average GC content of ORF sequences was 40.46%. About 42% of ORF sequences start with an initiation codon ATG, and 19.94% of ORF sequences had complete genomics with initiation and termination codon (Supplementary Figure 2). Bacteria, fungi, archaea, and phages were reported in all samples. Bacteria accounted for 99.37 percent of total gut symbionts, with fungi accounting for 0.63 percent. Archaea and phage proportions were low (Supplementary Table 3).

### The Composition of *Cnaphalocrocis medinalis* Gut Microbiota

**Figure 2:**
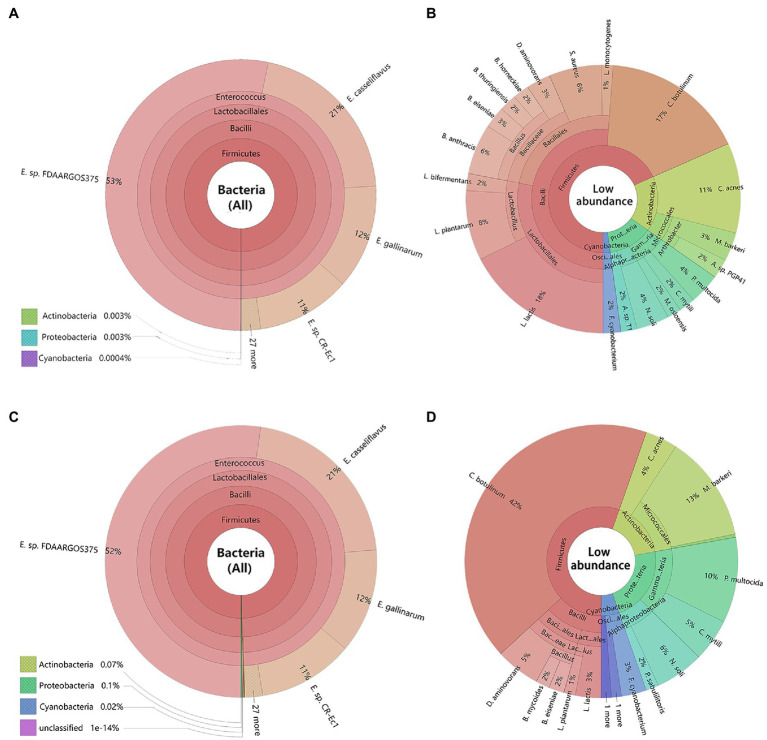
Composition of bacteria in the larvae gut of *Cnaphalocrocis medinalis* based on metagenomic data. The species were annotated using Krona. Different classification levels were indicated as circles by inside and outside. **(A)** All species of *C. medinalis* gut bacteria feeding with rice; **(B)** Low abundance species without *Enterococcus* of *C. medinalis* gut bacteria feeding with rice; **(C)** All species of *C. medinalis* gut bacteria feeding with maize seedling; and **(D)** Low abundance species without *Enterococcus* of *C. medinalis* gut bacteria feeding with maize seedling. The following website also has dynamic and more detailed information about Krona: https://licm.github.io/krona/rice-maize.html and https://licm.github.io/krona/krona2.html.

Metagenomics sequencing results revealed, the dominant bacteria in the gut of *C. medinalis* larvae were highly consistent between maize seedling feeding and rice feedings at all the different classification levels (Figures 2A,C). At the phylum level, Firmicutes were the most abundant bacteria in both rice and maize seedling feeding larvae *C. medinalis* (99.99%), followed by Actinobacteria, Proteobacteria, and Cyanobacteria. At class, order, and genus level, Bacilli, Lactobacillales, and *Enterococcus* sp. were the dominant bacteria (>99.99%) respectively. At the species level, gut bacteria of rice and maize seedling feeding larvae were mainly composed of *Enterococcus* as core dominant bacteria, including *E.* sp. FDAARGOS-375, *E. casseliflavus*, *E. gallinarum*, and *E.* sp. CR-Ec1, occupied about 97 and 96% in the gut of rice and maize group, respectively.

In addition, we analyzed the distribution of the rest of the bacteria except *Enterococcus*. After filtering out *Enterococcus*, there were great differences in composition and structure of the rest of the bacteria between rice and maize feeding larvae. The rice feeding larvae mainly composed of *Lactococcus* (18%), *Clostridium* (17%), *Bacillus* (13%), *Cutibacterium* (11%), and *Lactobacillus* (10%; Figure 2B), while the maize feeding larvae mainly composed of *Clostridium* (42%), *Microbacterium* (13%), and *Pasteurella* (10%, Figure 2D).

### Diversity and Enrichment Analysis

**Figure 3:**
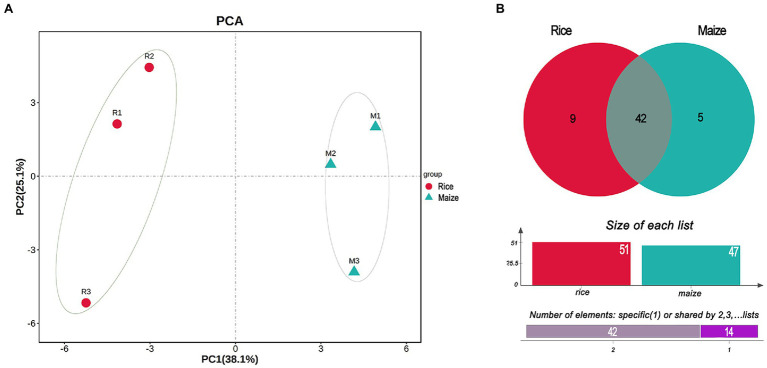
Principal component analysis (PCA) and Venn diagram analysis of the gut bacteria in *Cnaphalocrocis medinalis* fed with different diets. **(A)** Beta-diversity of the bacteria using principal coordinate analysis. **(B)** Venn diagram showing the number of shared or specific bacterial species.

The PCA plot of gut microbiota, calculated from the relative abundance of OTU, showed the difference between rice and maize feeding larvae in the profiles of the first component (PC1: 38.1%) and a second component (PC2: 25.1%). The results suggested that feeding with different diets had made a variation in the composition of gut microbiota (Figure 3A).

According to the Venn diagram analysis, 42 species of gut bacteria in *C. medinalis* were the same between rice and maize feeding groups, and 14 species were unique (Figure 3B). Among 14 unique species, nine species belonged to rice group and five species were unique to maize group. None of the 14 unique species were classified to *Enterococcus*. In contrast, among the shared 42 species, most of them were *Enterococcus*. Besides shared 31 strains of *Enterococcus* in both rice and maize groups, there were four strains shared from Bacilli, and the rest seven species belonged to Actinobacteria, Clostridia, Cyanobacteria, α-proteobacteria, and γ-proteobacteria (Supplementary Table 4). The shared taxonomic members might be regarded as the core microbiome of *C. medinalis* feeding on rice and maize seedling (Supplementary Table 4).

**Figure 4:**
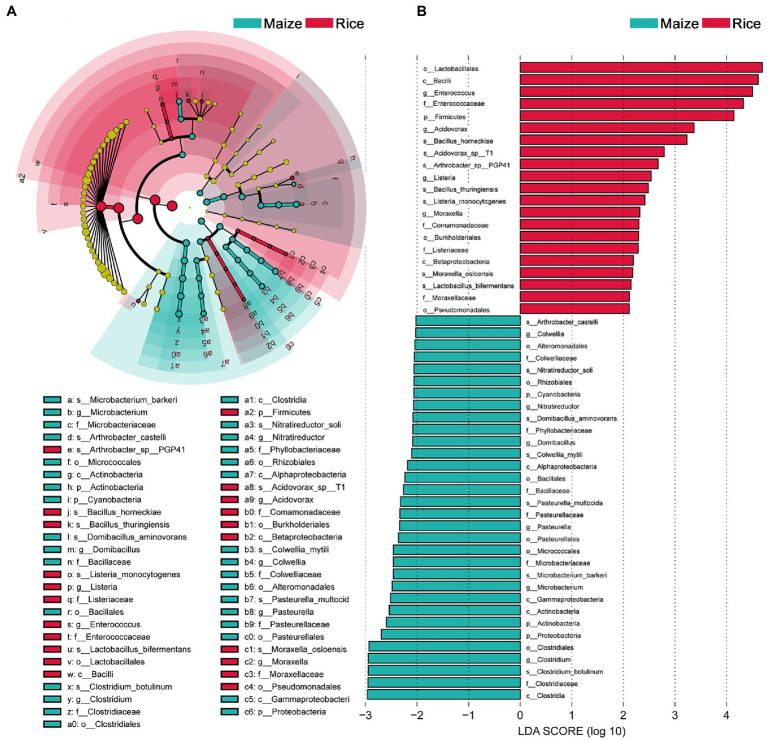
Comparison of microbial variations using the linear discriminant analysis effect size (LEfSe) online tool. **(A)** Cladogram for taxonomic representation of significant differences between treatments (*p* < 0.05). **(B)** Histogram of the LDA scores for differentially abundant features between treatments. The threshold on the logarithmic LDA score for discriminative features was set to 2.0. Differences are represented in the color of the most abundant taxa.

Linear discriminant analysis effect size analysis results showed that the community compositions between the two groups had significant differences. A total of 52 different abundant taxa were found to be enhanced in the rice and maize feeding group; these taxa could be used as biomarkers (LDA > 2, *p* < 0.05; Figure 4). The generic biomarkers in the rice group were Firmicutes at phylum level, while Cyanobacteria, Actinobacteria, and Proteobacteria played an important role in the maize feeding group. There had been 10 distinguishable different enrichment families between the two groups, with Enterococcaceae, Comamonadaceae, Listeriaceae, and Moraxellaceae likely played important roles in rice feeding larval gut, Colwelliaceae, Phyllobacteriaceae, Bacillaceae, Pasteurellaceae, Microbacteriaceae, and Clostridiaceae enriched in maize feeding larval gut. A total of 14 species significantly enriched, *B. horneckiae*, *Acidovorax* sp. T1, *Arthrobacter* sp. PGP41, *B. thuringiensis*, *Listeria monocytogenes*, *Moraxella osloensis*, and *L. bifermentans* exhibited a relatively higher abundance in rice feeding larvae gut, and *Clostridium botulinum*, *M. barkeri*, *P. multocida*, *Colwellia mytili*, *Domibacillus aminovorans*, *Nitratireductor soli*, and *A. castelli* were relatively more abundant in maize feeding larval gut.

### Functional Analysis of *Cnaphalocrocis medinalis* Gut Microbes

**Figure 5:**
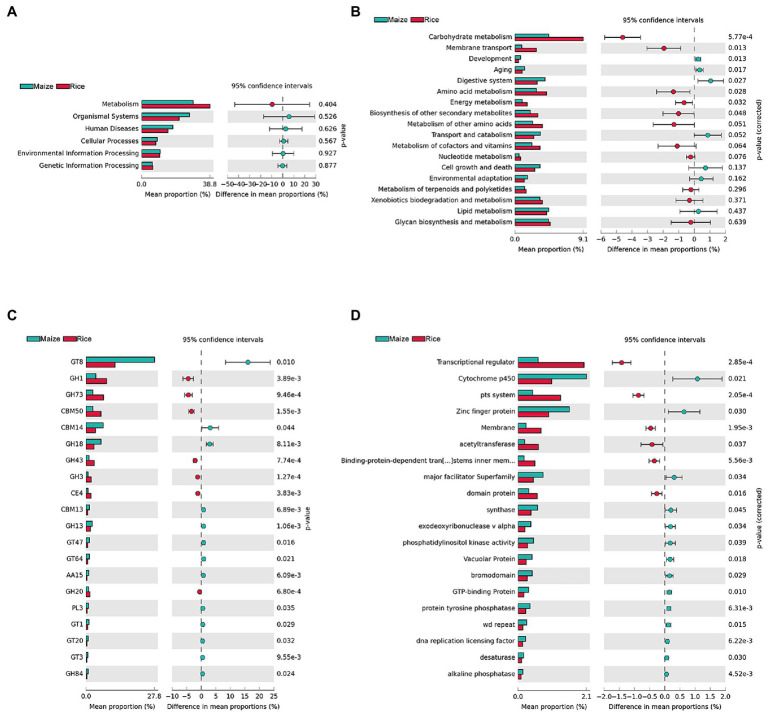
Inferred functions of gut microbes in rice and maize feeding *Cnaphalocrocis medinalis*. **(A)** KEGG level 1 analysis, **(B)** KEGG level 2 analysis, **(C)** CAZy analysis (top 20 enriched carbohydrate-active enzymes), and **(D)** eggNOG analysis (top 20 enriched functions).

KEGG, CAZy, and eggNOG were used to annotate gene functions (Figure 5). STAMP was applied to compare the relative abundance values for further investigating whether there was a significant difference between different feeding populations. The results showed that there were no significant differences in the first level of KEGG pathways (metabolism, organismal systems, human diseases, cellular processes, environmental information processing, and genetic information processing) between the two groups, and the metabolism was the most abundant function (representing 38.80 ± 15.48 and 29.31 ± 5.47% in the gut microbiota of the rice and maize feeding *C. medinalis*, *p* = 0.404; Figure 5A). For additional investigation, we looked at the second level of KEGG pathways. A total of eight functions (Carbohydrate metabolism, membrane transport, development, aging, digestive system, amino acid metabolism, energy metabolism, and biosynthesis of other secondary metabolites) showed significant differences (*p* < 0.05; Figure 5B). Carbohydrate metabolism was the most enriched function in both groups and was more abundant in rice feeding than maize feeding group. In addition, membrane transport, amino acid metabolism, energy metabolism, and biosynthesis of other secondary metabolites were more abundant in the rice feeding group, while the functions of growth, aging, and the digestive system, on the other hand, were all higher in the maize-fed group.

*Cnaphalocrocis medinalis* gut microbes were rich in carbohydrate degradation-related genes. CAZyme analysis found 92 carbohydrate-active enzymes (CAZymes), including 38 glycoside hydrolases (GHs), 29 glycosyltransferases (GTs), nine carbohydrate-binding modules (CBMs), seven carbohydrate esterases (CEs), five polysaccharide lyases (PLs), and four auxiliary activities (AAs; Supplementary Table 5). The top 20 enriched CAZymes with significant differences were shown in Figure 5C. GH1, GH73, CBM50, GH43, GH3, CE4, and GH20 were significantly higher in the rice group than in the maize group, while GT8, CBM14, GH18, and other 10 types of CAZymes were significantly higher in the maize group, implying that the two groups might have different carbohydrate metabolism pathways.

The eggNOG analysis results showed that there were a large number of significant functional differences between rice and maize feeding groups, such as Transcriptional regulator, Cytochrome p450, pts. system, and Zinc finger protein, etc. Figure 5D showed the top 20 enriched eggNOG functions with significant differences between rice and maize feeding *C. medinalis* groups.

### Biodegradation of Carbohydrates

A string of carbohydrate metabolism-related enzyme-encoding genes was identified by KEGG analysis from the gut microbe metagenomic of rice and maize feeding *C. medinalis* (Supplementary Table 6). Catalase, catalase-peroxidase, and glutathione peroxidase were found to be involved in lignin degradation when compared to the lignocellulose degradation pathways. In the biodegradation of cellulose and hemicellulose, 17 genes were found to be involved, including cellulose synthase, glucosidase, galactosidase, and others. For xylan and pectin biodegradation, endo-1,4-beta-xylanase, xylan 1,4-beta-xylosidase, and pectinesterase were identified. Starch and sucrose metabolic functional prediction showed there were 62 genes involved in the pathway (Supplementary Figure 3). The gut bacteria of *C. medinalis* had a complete metabolic pathway that degraded starch and sucrose into D-glucose.

### Amino Acid Synthesis and Metabolism of Vitamin B6

Based on the amino acid metabolic pathway analysis, *C. medinalis* gut bacteria metagenome possessed the complete pathway for biosynthesis of Arginine, Lysine, Valine leucine, and Isoleucine (Supplementary Figures 4–6). The gut bacteria also participated in the metabolism of vitamin B6. The enzymes involved in vitamin B6 metabolism pathway mainly included pyridoxine kinase (pdxK), pyridoxamine 5′-phosphate oxidase (pdxH), pyridoxal phosphatase (pdxP), and pyridoxal phosphate phosphatase PHOSPHO2 (PHOSPHO2), etc. (Supplementary Figure 7).

### Functional Analysis of the *Enterococcus*

**Figure 6:**
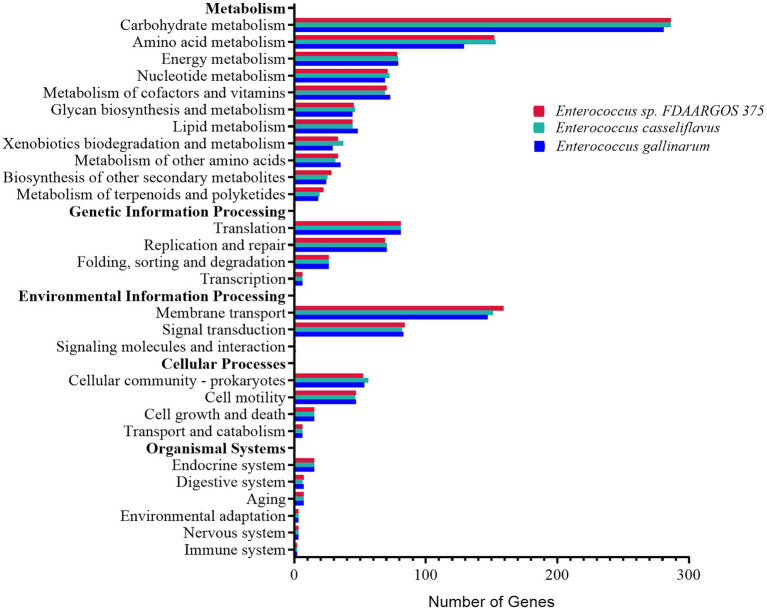
Functional distribution analysis of genes by the KEGG main categories, from the genomes of the three most abundant *Enterococcus* species, *Enterococcus* sp. FDAARGOS-375, *Enterococcus casseliflavus*, and *Enterococcus gallinarum*.

The distribution of KEGG categories was similar among the three most abundant *Enterococcus* species, *E.* sp. FDAARGOS-375, *E. casseliflavus*, and *E. gallinarum* in both rice and maize feeding groups (Figure 6). The genes associated with metabolism (862, 861, and 829 genes, respectively) and environmental information processing (244, 234, and 231 genes, respectively) were abundant. The major pathways were associated with carbohydrate metabolism, membrane transport, and amino acid metabolism. Furthermore, the three *Enterococcus* species all possessed 14 complete pathways of carbohydrate metabolism, including galacturonate degradation, galactose degradation, and glycogen biosynthesis, etc., biosynthesis pathways of 11 amino acids, and can synthesize vitamins, including riboflavin (vitamin B2) and menaquinone (vitamin K2; Supplementary Table 7). It was implied that the core dominant species of *Enterococcus* contributed an essential role to the nutrition intake and development of *C. medinalis*.

## Discussion

Due to the great difficulty in achieving aseptic rearing conditions, many studies use the method of incorporating antibiotics into diet to determine the biological function of gut bacteria (Ignasiak and Maxwell, 2018; Koskinioti et al., 2019; Duplouy et al., 2020; Duan et al., 2021). The nutrition indices of *RGR*, *RCR*, *ECI*, and *ECD* were used to estimate the effect of food utilization. *RGR* and *RCR* represent the efficiency of food digestion and subsequent conversion to body mass (Slansky and Wheeler, 1991), while *ECI* and *ECD* reflect the conversion rates of food ingested and digested into body biomass (Nathan et al., 2005; Chen et al., 2020). Antibiotic treatment eliminating gut bacteria significantly reduced the *RGR* and *RCR* of *S. litura* larvae (Xia et al., 2020). Gut microbes helped *Drosophila melanogaster* larvae develop faster by improving the host’s nutrition and metabolism compared to antibiotic treatment (Ridley et al., 2012). Similarly, the present study showed that culturable bacteria from the intestinal tract of *C. medinalis* were eliminated effectively combining use of antibiotics. Thus, resulted into negative effect on the food utilization indices of *C. medinalis*, the nutrition indices significantly decreased after antibiotic treatement. It was evidenced that the microbiota loading in the gut were essential for the larva growth and development of *C. medinalis*.

Insect gut microbiomes differ between and within-host species and they often have multiple effects on insect biology (Douglas, 2015; Voirol et al., 2018). To gain more insight into the interactions between symbiotic microorganism and their insect hosts, we need to study the gut bacterial diversity firstly (Duplouy et al., 2020). In the present study, the diversity of gut bacteria in the fourth instar larvae of *C. medinalis* was analyzed by metagenomic sequencing. A total of 56 species bacteria were identified in the rice and maize feeding groups, belonging to 20 genera, 19 families, 13 orders, seven classes, and four phyla. In general, Firmicutes in phylum level, *Enterococcus* in genus level constituted the largest proportion. Yang et al. (2020) found that the gut bacteria of *C. medinalis* during the later larval stages were dominated by *Enterococcus* and Enterobacteriaceae-unclassified, this was similar but not identical with our findings, which might be linked with the different feeding environments (Staudacher et al., 2016; Mason et al., 2019, 2021). Though the dominant bacteria in the gut of *C. medinalis* larvae were highly consistent between rice and maize feedings groups at classification levels, PCA analyses showed that different diets had made a variation in the composition of gut microbiota, there were 14 unique species belonged to different feeding groups, and a total of 52 different abundant taxa were found enriched in the two feeding groups. It was indicated that different diets had caused a variation in gut microbiota composition. The gut microbiota were in part driven by the diet provided.

Whether there are resident bacteria in the intestinal tract of herbivorous insects remains unsettled. By some studies, there were specific gut bacterial species that occurred regardless of what their host insect feed upon (Robinson et al., 2010; Pinto-Tomás et al., 2011; Xia et al., 2013), whereas others showed caterpillars lack resident, host insect-specific, and food-independent bacteria, therefore, food might have a significant influence on bacterial community variability (Staudacher et al., 2016; Hammer et al., 2017). We experimented with rice and maize seedlings to compare the bacterial community compositions between different feeding diets. Results showed that the dominant bacteria in the two feeding groups were highly consistent in the gut of fourth instar larvae of *C. medinalis*, with the gut bacterial community dominated by Firmicutes at the phylum level and *Enterococcus* at the genus level, mainly including *E.* sp. FDAARGOS-375, *E. casseliflavus*, *E. gallinarum*, and *E.* sp. CR-Ec1. Firmicutes were particularly strongly represented gut bacterial in insect gut based on the analysis of 218 insect species in 21 taxonomic orders (Yun et al., 2014). *Enterococcus* was one of the most widespread bacteria at the genus level which was presented in more than 70% of the studied lepidopteran species (Vistto et al., 2009; Schmid et al., 2014; Chen et al., 2016; Voirol et al., 2018). As the core dominant gut bacteria of *C. medinalis* found in the present study, *Enterococcus* also revealed as domination bacterial groups in second and thirrd instar larval gut of *C. medinalis* in the other two studies (Yang, 2012; Yang et al., 2020). This indicated the larvae of *C. medinalis* might maintain essentially groups of bacteria residing in the gut. The results in the present study were not entirely consistent with the previous studies to some other lepidopteran insects, *L. xylina*, *S. litura*, and *H. armigera*, with which the dominant bacteria were significantly affected by different diets (Priya et al., 2012; Xia et al., 2020; Ma et al., 2021). This difference might result from different feeding habits, *L. xylina*, *S. litura*, and *H. armigera* are all highly polyphagous, while *C. medinalis* is oligophagous, and the larvae are almost only exclusively in rice. It is therefore possible that independence of symbiosis might have facilitated switching to different host plants and promoted diversification (Voirol et al., 2018).

Gut microbiota play important roles in host nutrition, given the limited digestion capacity of lepidopteran insect intestines (Engel and Moran, 2013; Jang and Kikuchi, 2020; Wang et al., 2020). Due to most of the gut bacteria are non-culturable for now; metagenomic sequencing technology provides a new possibility to predict the potential functions of the gut microbiota (Chen et al., 2019). Carbohydrates are a significant source of energy for life-sustaining activities. We identified a series of carbohydrate-active enzymes in *C. medinalis* gut symbiotic bacteria, and it might be involved in cellulose and carbohydrate degradation pathways, including biodegradation of lignin, cellulose, hemicellulose, xylan, and pectin, as well as a complete metabolic pathway that degrades starch and sucrose into D-glucose. We also found the genes of the most abundant three *Enterococcus* species in *C. medinalis* gut symbiotic bacteria, *E.* sp. FDAARGOS-375, *E. casseliflavus*, and *E. gallinarum* were major associated with the carbohydrate metabolism KEGG metabolic pathway, 14 complete central, and other carbohydrate metabolism pathways were found, including galacturonate degradation, galactose degradation, and glycogen biosynthesis, etc. *Enterococcus casseliflavus* was reported to form a bacterial biofilm in the gut of *S. litura* and *Hyles euphorbiae* and contribute to the immobilization of latex-like molecules in the larvae (Shao et al., 2011; Vilanova et al., 2016). Similarly, Xia et al. found there were significant numbers of genes that encoded carbohydrate-active enzymes in the *P. xylostella* and *S. litura* gut microbiome (Xia et al., 2017, 2020). Herbivorous insects require plant cell wall degrading enzymes (PCWDEs), such as cellulases, hemicellulases, and pectinases to break down plant cell walls in order to absorb carbohydrates. Previous studies have shown that the genes encoding PCWDEs were lacking in most of the studied Lepidoptera, and lepidopteran species might rely on symbionts for cellulose digestion (Xia et al., 2017; Voirol et al., 2018).

Another advantage that flows from gut microbes is that they could provide additional nutrition, such as amino acids and vitamins (Wang et al., 2020). The entire biosynthesis pathway for arginine, lysine, valine, leucine, and isoleucine, as well as vitamin B2, B6, and K2 biosynthesis cooperation, were found in the *C. medinalis* gut bacteria metagenome. Although many studies have linked symbiotic bacteria to amino acid and vitamin supplementation (Brune and Dietrich, 2012; Salem et al., 2014; Ayayee et al., 2015; Douglas, 2015), it is still unclear whether these amino acids and vitamins are secreted into the host intestine. Our results only showed the potential functions of the gut microbes, further research is needed to identify these functions.

In conclusion, our results indicated that microbiota loading in the gut were essential for the larva growth and development of *C. medinalis*, and different diets influenced the changes in larvae gut microbiota. Coupled with recent work, the gut microbes associated with *C. medinalis* were not simply transient associates and diet cannot fully explain variation observed in the gut microbiome, suggested that the larvae of *C. medinalis* hosted a core dominant community of *Enterococcus*, which was independent from the diets. Gut microbiota especially the dominant *Enterococcus* species had the functions of carbohydrates biodegradation, amino acids, and vitamins synthesis; therefore, *C. medinalis* may rely on the gut microbiota to help it get nutrients. However, the exact mechanisms of gut bacteria participation in these roles remain unclear, requiring further study for verification. Our work provides a perspective and direction for further study on the functions of the gut microbiota of the important pest species *C. medinalis*.

## Data Availability Statement

The datasets presented in this study can be found in online repositories. The names of the repository/repositories and accession number(s) can be found in the article/Supplementary Material.

## Author Contributions

CL and JX designed the project, analyzed the data, and wrote and edited the manuscript. GH, JS, and LH contributed to the methodology and data curation. YL, YX, and QL conducted the experiment and collected the samples. All authors contributed to the article and approved the submitted version.

## Funding

This work was supported by the project of the Natural Science Foundation of Jiangsu Province, China (grant no. BK20191216), the International Cooperation Project of Jiangsu Province (grant no. BZ2020039), the National Agricultural Basic Long-Term Scientific and Technological Work (NAES069AM04 and NAES-EE-041), the Jiangsu Agricultural Science and Technology Innovation Fund [grant no. CX(20)1004], and Yangzhou Science and Technology Project (grant nos. YZ2021049 and YZ2021034).

## Conflict of Interest

JS and QL are employed by Yangzhou Luyuan Bio-Chemical Co., Ltd., Yangzhou, China.

The remaining authors declare that the research was conducted in the absence of any commercial or financial relationships that could be construed as a potential conflict of interest.

## Publisher’s Note

All claims expressed in this article are solely those of the authors and do not necessarily represent those of their affiliated organizations, or those of the publisher, the editors and the reviewers. Any product that may be evaluated in this article, or claim that may be made by its manufacturer, is not guaranteed or endorsed by the publisher.

## Footnotes

^1^ https://www.r-project.org/
