# Supplementary material for: The Gut Microbiota Composition of Cnaphalocrocis medinalis and Their Predicted Contribution to Larval Nutrition
Source: Front Microbiol. 2022 May 20;13:909863. doi: 10.3389/fmicb.2022.909863 (PMC9166232; doi:10.3389/fmicb.2022.909863)
Supplement: Supplementary file 1 [file Data_Sheet_1.docx]

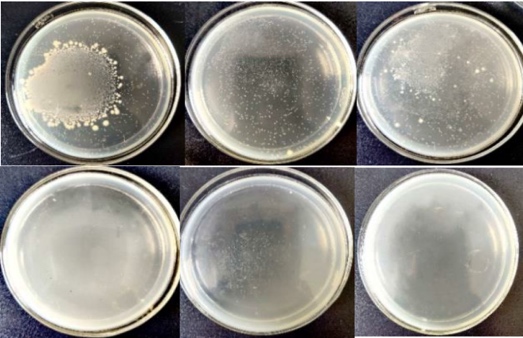


**A**

**B**

**Supplementary FIGURE 1 Cultures of gut bacteria after treated with combining antibiotics.** (A) Control group; (B) Treated group

**
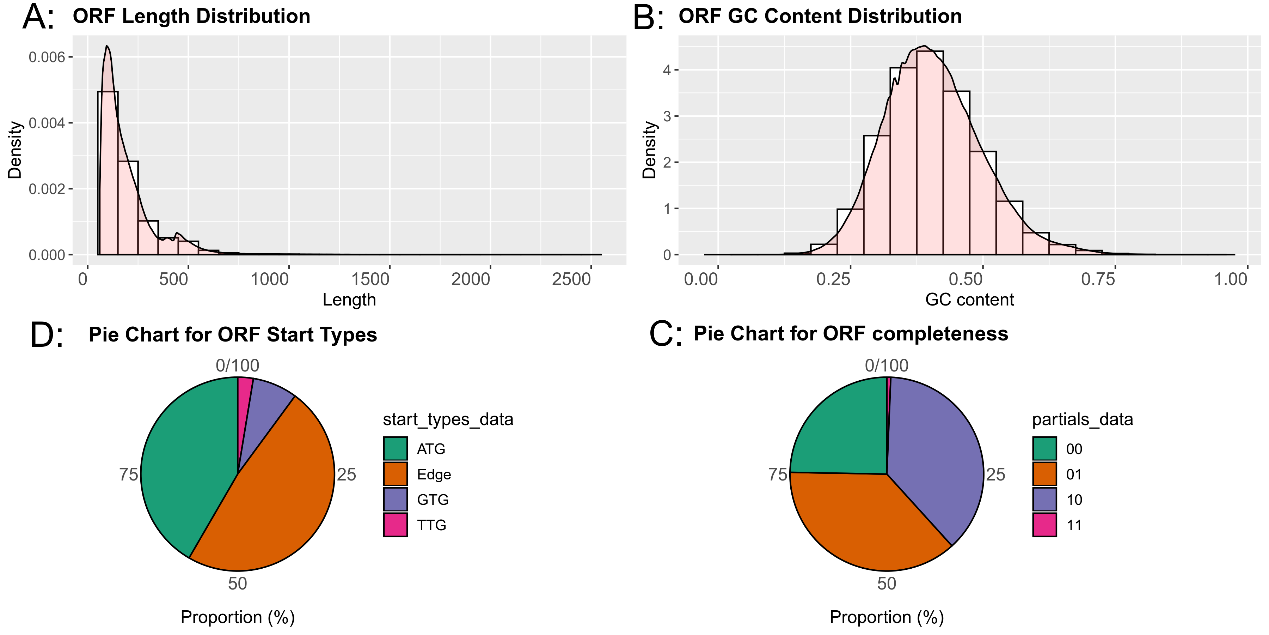
**

**Supplementary FIGURE 2 Gene prediction of the gut microbiota metagenome of *C. medinalis*.** (A) Histogram of length distribution of predictive genes; (B) Histogram of the percentage of GC content in the bases of predictive genes; (C) Pie chart for ORF start types, Edge represents the unknown initiation codon genes; (D) Pie chart for ORF completeness. 00-complete genomics with initiation and termination codon; 01-genomics with only initiation codon; 10-genomics with only termination codon; 11- genomics with no initiation or termination codon.

**
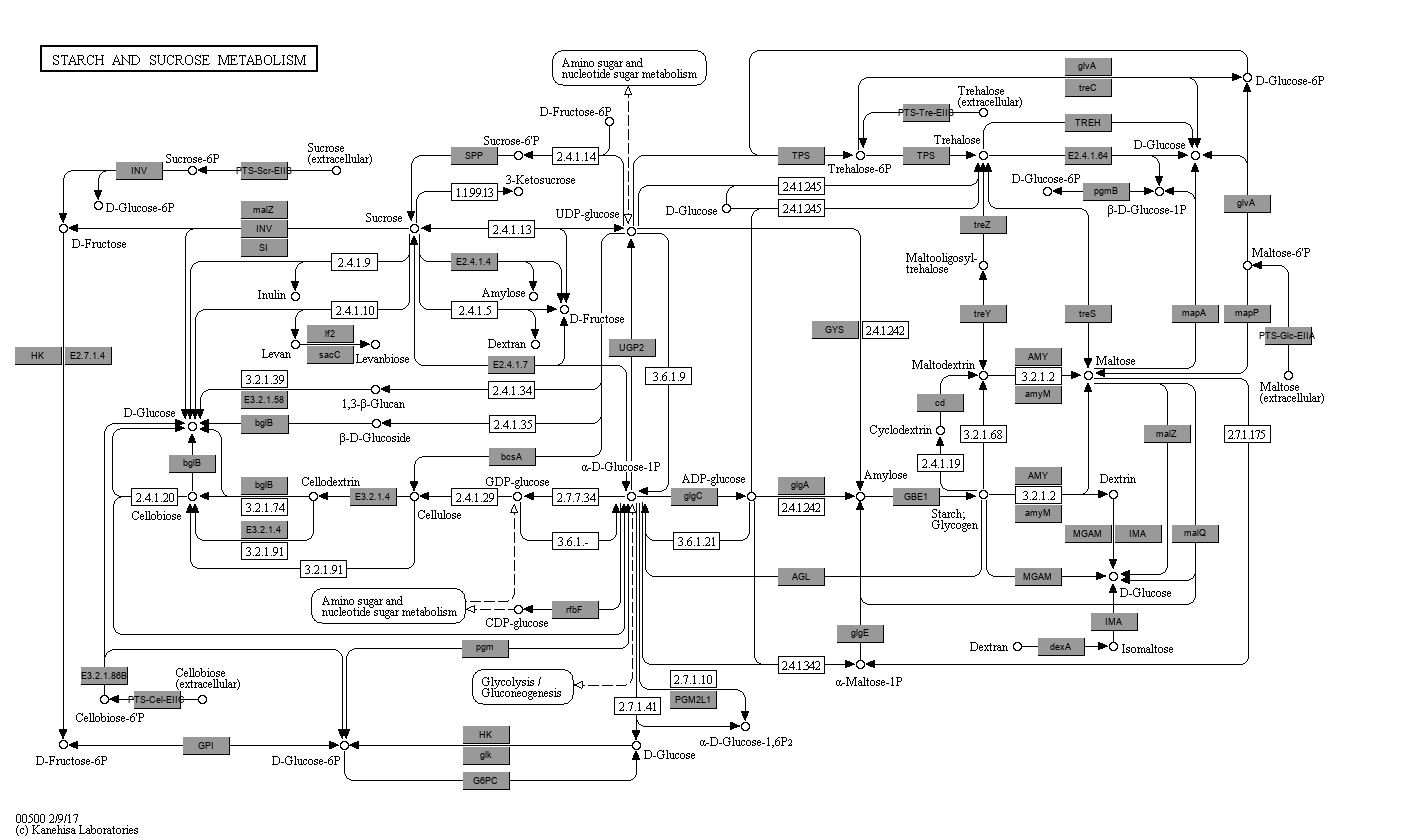
**

**Supplementary FIGURE 3** Starch and sucrose metabolic pathway of *C. medinalis* gut bacteria.

Gray squares represent the genes were finding in the intestinal metagenome.

**
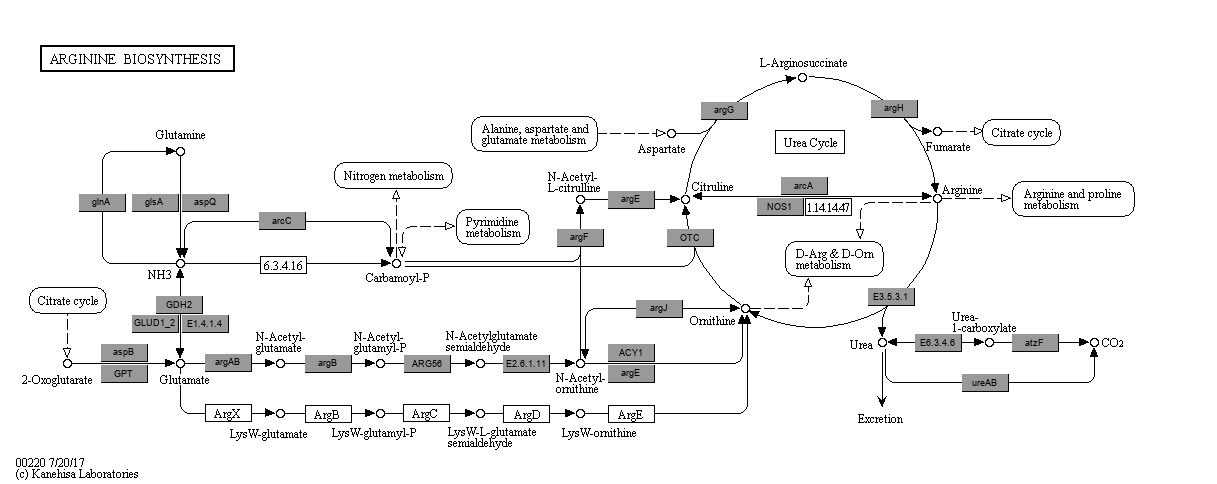
**

**Supplementary FIGURE 4** Arginine biosynthesis pathway of *C. medinalis* gut bacteria.

**
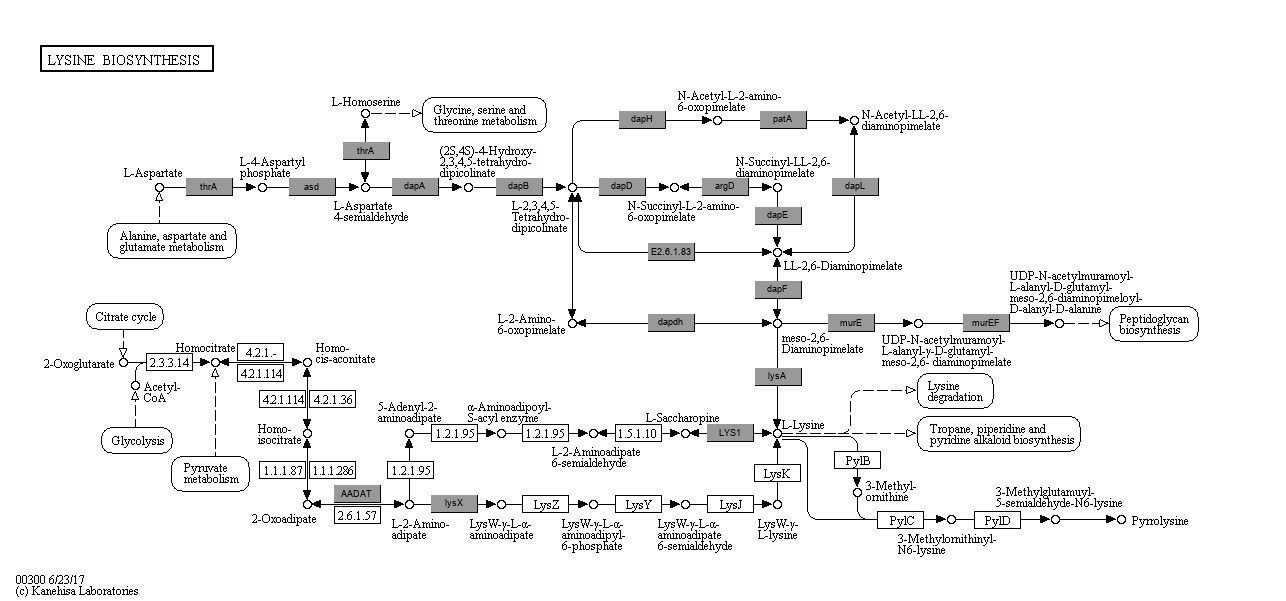
**

**Supplementary FIGURE 5** Lysine biosynthesis pathway of *C. medinalis* gut bacteria.

**
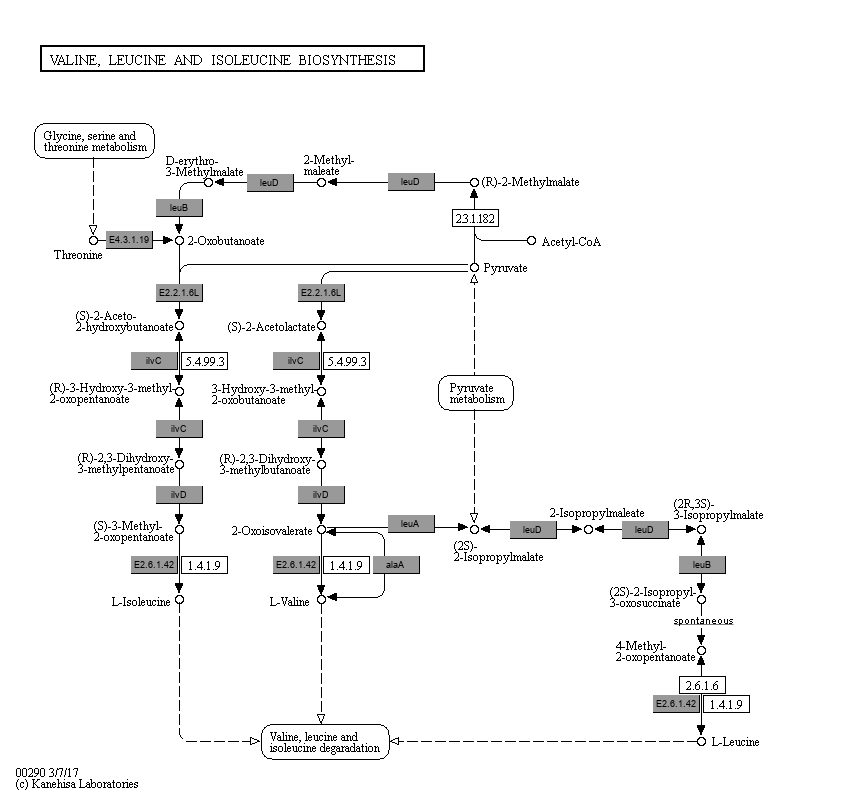
**

**Supplementary FIGURE 6** Valine, Leucine and Isoleucine biosynthesis pathway of *C. medinalis* gut bacteria.

**
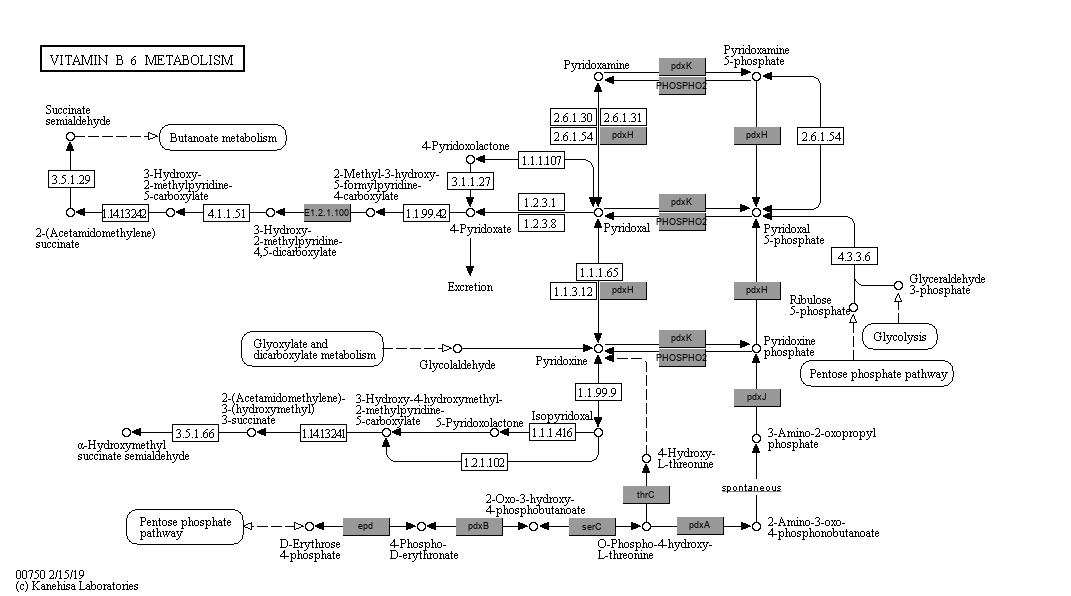
**

**Supplementary FIGURE 7** Vitamin B6 metabolism pathway of *C. medinalis* gut bacteria.
