# Supplementary material for: The Gut Microbiota Composition of Cnaphalocrocis medinalis and Their Predicted Contribution to Larval Nutrition
Source: Front Microbiol. 2022 May 20;13:909863. doi: 10.3389/fmicb.2022.909863 (PMC9166232; doi:10.3389/fmicb.2022.909863)
Supplement: Supplementary file 2 [file Table_1.DOCX]

**Supplementary Table 1** Numerical data of the gut microbiota metagenome of *C. medinalis*

| **Sample ID** | **InsertSize(bp)** | **RawReads**  **(#)** | **RawBase**  **(GB)** | **%GC** | **Clean reads**  **(#)** | **Cleaned (%)** | **Clean Q20 (%)** | **Clean Q30 (%)** |
| --- | --- | --- | --- | --- | --- | --- | --- | --- |
| Rice-1 | 350 | 23025739 | 6.91 | 42 | 21280081 | 92.42 | 98.39 | 94.09 |
| Rice-2 | 350 | 22549144 | 6.76 | 42 | 20788640 | 92.19 | 98.43 | 94.21 |
| Rice-3 | 350 | 22905432 | 6.85 | 42 | 21354218 | 93.23 | 98.56 | 94.35 |
| Maize-1 | 350 | 22238108 | 6.67 | 40 | 20541916 | 92.37 | 98.43 | 94.21 |
| Maize-2 | 350 | 24272783 | 7.28 | 39 | 22516148 | 92.76 | 98.48 | 94.36 |
| Maize-3 | 350 | 24167998 | 7.25 | 38 | 22427976 | 92.80 | 98.48 | 94.33 |

Sample ID: sample name; InsertSize (bp): represents the use of a 350 bp Library; RawReads: sequencing number of raw reads; RawBase: total base pair number in the sequencing raw data, calculated by the product of RawReads and the sequence length; %GC: the percentage of GC content in the bases; Clean reads: the valid reads number after filtered; Cleaned (%): the percentage of Cleanreads to Rawreads. Clean Q20, Q30: the percentage of base numbers in CleanData with a mass value greater than 20 and 30.

**Supplementary Table 2** Assembly analysis of the gut microbiota metagenome of *C. medinalis*

| **Size of contigs** | **Number of contigs** | **Total length（Mb）** |
| --- | --- | --- |
| >=0bp | 3323969 | 2955.93 |
| >=1000bp | 786263 | 1205.23 |
| >=5000bp | 5609 | 42.79 |
| >=10000bp | 363 | 10.68 |
| >=25000bp | 103 | 6.85 |
| >=50000bp | 47 | 4.88 |
| Largest contig (bp) | 328345 | |
| N50 (bp) | 882 | |
| N75 (bp) | 667 | |
| GC (%) | 38.52 | |

Number of contigs: the total number of Contigs assembled; Total length (Mb): the total length of Contigs assembled; N50, N75: Contigs are sorted by length from long to short, when the sum value reaches 50% or 75% of the total length of Contigs.

**Supplementary Table 3** Sequence number of different species in the *C. medinalis* gut

|  | Bacteria | Fungi | Archaea | Phages |
| --- | --- | --- | --- | --- |
| Sequence Number | 5641600 | 35725 | 25 | 36 |

**Supplementary Table 4** Common and specific gut bacteria species in the rice and maize feeding groups

| common species | | | |
| --- | --- | --- | --- |
| *Enterococcus_aquimarinus* | *Enterococcus_hermanniensis* | | *Enterococcus_sulfureus* |
| *Enterococcus_asini* | *Enterococcus_hirae* | | *Enterococcus_thailandicus* |
| *Enterococcus_canis* | *Enterococcus_italicus* | | *Enterococcus_wangshanyuanii* |
| *Enterococcus_casseliflavus* | *Enterococcus_malodoratus* | | *Pasteurella_multocida* |
| *Enterococcus_cecorum* | *Enterococcus_massiliensis* | | *Bacillus_eiseniae* |
| *Enterococcus_columbae* | *Enterococcus_mundtii* | | *Clostridium_botulinum* |
| *Enterococcus_devriesei* | *Enterococcus_pallens* | | *Colwellia_mytili* |
| *Enterococcus_dispar* | *Enterococcus_phoeniculicola* | | *Cutibacterium_acnes* |
| *Enterococcus_durans* | *Enterococcus_pseudoavium* | | *Domibacillus_aminovorans* |
| *Enterococcus_faecalis* | *Enterococcus_rivorum* | | *Filamentous_cyanobacterium* |
| *Enterococcus_faecium* | *Enterococcus_saccharolyticus* | | *Lactobacillus_plantarum* |
| *Enterococcus_gallinarum* | *Enterococcus_sp._CR-Ec1* | | *Lactococcus_lactis* |
| *Enterococcus_gilvus* | *Enterococcus_sp._FDAARGOS_375* | | *Microbacterium_barkeri* |
| *Enterococcus_haemoperoxidus* | *Enterococcus_sp._FDAARGOS_553* | | *Nitratireductor_soli* |
| specific species | | | |
| Rice feeding group | | Maize feeding group | |
| *Arthrobacter_sp._PGP41* | | *Arthrobacter_castelli* | |
| *Bacillus_thuringiensis* | | *Bacillus_mycoides* | |
| *Moraxella_osloensis* | | *Pseudoruegeria_sabulilitoris* | |
| *Staphylococcus_aureus* | | *Crinalium_epipsammum* | |
| *Bacillus_anthracis* | | *Synechococcus_sp._JA-3-3Ab* | |
| *Lactobacillus_bifermentans* | |  | |
| *Acidovorax_sp._T1* | |  | |
| *Bacillus_horneckiae* | |  | |
| *Listeria_monocytogenes* | |  | |

**Supplementary Table 5** Relative Abundance of carbohydrate active enzymes in gut microbes of *C. medinalis*.

|  | R1 | R2 | R3 | M1 | M2 | M3 |
| --- | --- | --- | --- | --- | --- | --- |
| AA1 | 21.80346 | 20.36377 | 22.375328 | 16.77422 | 23.30888 | 22.084736 |
| AA10 | 43.15128 | 42.46365 | 40.212474 | 14.61319 | 18.86943 | 19.323662 |
| AA15 | 11.73397 | 9.96917 | 10.585092 | 17.28348 | 16.31869 | 18.068951 |
| AA3 | 18.04533 | 20.2029 | 19.489863 | 12.70591 | 14.928 | 18.524 |
| CBM1 | 0.20234 | 0.19535 | 0.245639 | 0 | 0 | 0 |
| CBM12 | 15.91819 | 16.50208 | 16.882846 | 4.6492 | 4.19381 | 3.464082 |
| CBM13 | 16.52056 | 17.38134 | 14.932301 | 20.63674 | 24.77405 | 19.806057 |
| CBM14 | 103.57792 | 110.95677 | 94.349633 | 118.69057 | 121.35799 | 78.479317 |
| CBM39 | 3.0603 | 2.87037 | 3.040536 | 2.17207 | 1.97457 | 2.701121 |
| CBM47 | 4.95451 | 4.93343 | 4.835826 | 6.73849 | 6.3166 | 3.888436 |
| CBM48 | 18.9153 | 17.57199 | 20.102976 | 9.50622 | 6.58611 | 8.325813 |
| CBM50 | 180.0666 | 154.35044 | 157.405954 | 39.47258 | 42.66699 | 41.664352 |
| CBM61 | 14.03438 | 14.07529 | 15.226746 | 2.23876 | 2.38816 | 3.430114 |
| CE0 | 15.94768 | 17.56895 | 15.254709 | 15.13357 | 14.07624 | 20.077772 |
| CE1 | 38.58766 | 38.22109 | 30.156472 | 8.6571 | 8.45541 | 5.836083 |
| CE12 | 18.58667 | 21.2552 | 20.448458 | 3.77038 | 4.14941 | 6.023181 |
| CE4 | 55.20338 | 48.60665 | 57.975857 | 11.72646 | 14.31984 | 10.627503 |
| CE7 | 18.84311 | 16.03398 | 18.392449 | 3.71685 | 4.74684 | 3.441348 |
| CE8 | 15.35257 | 13.54744 | 15.887023 | 3.17425 | 3.42734 | 2.43393 |
| CE9 | 19.08211 | 15.75188 | 17.35656 | 6.20298 | 7.21678 | 6.361142 |
| GH1 | 211.00765 | 222.16622 | 246.409905 | 57.99356 | 71.17942 | 52.118886 |
| GH10 | 0.47331 | 0.50281 | 0.481502 | 1.32195 | 1.24448 | 1.844617 |
| GH105 | 14.80692 | 13.59136 | 14.737868 | 1.48393 | 1.65615 | 1.889384 |
| GH116 | 0.32556 | 0.38895 | 0.349131 | 0.48644 | 0.48903 | 0.358642 |
| GH123 | 14.45977 | 13.83131 | 16.138275 | 2.28492 | 2.25181 | 3.620468 |
| GH125 | 14.71637 | 14.43175 | 13.143183 | 3.0684 | 3.61681 | 2.46242 |
| GH127 | 14.97074 | 15.57096 | 16.240597 | 4.56179 | 3.70593 | 3.555457 |
| GH13 | 45.67823 | 49.296 | 41.220523 | 40.92122 | 39.31887 | 33.853741 |
| GH133 | 5.2023 | 5.93753 | 5.962096 | 7.86178 | 7.03941 | 5.576072 |
| GH16 | 9.72251 | 9.67515 | 10.525837 | 8.81916 | 9.1365 | 6.90399 |
| GH18 | 90.45775 | 83.77062 | 83.614571 | 87.10903 | 101.34462 | 89.750933 |
| GH19 | 1.7948 | 1.74473 | 1.973177 | 3.40229 | 4.49787 | 3.717577 |
| GH20 | 43.93539 | 42.29584 | 39.637424 | 15.9856 | 15.7094 | 13.943838 |
| GH23 | 21.14191 | 20.39349 | 19.947701 | 3.27954 | 3.78961 | 3.026618 |
| GH27 | 30.54288 | 29.33681 | 27.661033 | 19.9492 | 18.58672 | 13.80292 |
| GH28 | 13.67067 | 14.74191 | 16.494519 | 2.9542 | 2.21012 | 2.233251 |
| GH29 | 42.62564 | 47.92368 | 42.026527 | 11.16051 | 9.07371 | 11.880393 |
| GH3 | 53.6453 | 57.74764 | 55.693635 | 13.83834 | 11.97982 | 10.733528 |
| GH31 | 33.37332 | 32.57832 | 29.369916 | 9.72887 | 11.04519 | 9.802741 |
| GH32 | 23.66613 | 24.81147 | 28.931619 | 5.4491 | 4.99305 | 5.370289 |
| GH35 | 28.30055 | 27.31135 | 30.179136 | 6.36308 | 7.03009 | 7.265985 |
| GH36 | 13.62878 | 12.71132 | 13.144382 | 3.43324 | 2.74013 | 2.438475 |
| GH37 | 22.36953 | 23.18816 | 17.703592 | 18.66992 | 19.05399 | 11.478454 |
| GH38 | 40.78324 | 45.31214 | 37.848741 | 10.32019 | 11.8785 | 8.641025 |
| GH4 | 46.63945 | 44.88703 | 39.411991 | 8.40284 | 8.43966 | 8.915161 |
| GH42 | 58.66708 | 57.03316 | 45.795181 | 9.83137 | 12.83731 | 11.336734 |
| GH43 | 88.29207 | 97.09807 | 79.586717 | 19.28201 | 20.6881 | 13.824084 |
| GH46 | 1.39048 | 1.31903 | 1.638093 | 1.07588 | 1.04626 | 1.697718 |
| GH47 | 13.45702 | 12.67501 | 12.304819 | 14.0788 | 12.34879 | 10.487983 |
| GH53 | 15.34111 | 14.82667 | 13.168636 | 2.25743 | 3.01878 | 2.780824 |
| GH6 | 1.31161 | 1.49266 | 1.543333 | 1.44504 | 1.04022 | 1.334598 |
| GH65 | 26.52276 | 26.47121 | 31.312585 | 5.90618 | 5.9528 | 9.138038 |
| GH73 | 197.86256 | 173.83348 | 200.630784 | 33.57183 | 46.99978 | 42.307217 |
| GH78 | 11.65133 | 11.30201 | 12.645497 | 1.75705 | 1.74661 | 2.591368 |
| GH84 | 12.26997 | 12.18863 | 9.479773 | 14.27378 | 10.86927 | 13.73854 |
| GH88 | 16.26507 | 16.71084 | 17.428425 | 3.94219 | 3.77955 | 4.12505 |
| GH92 | 13.56119 | 14.33187 | 12.649081 | 3.36229 | 2.61684 | 2.869589 |
| GH93 | 0.21641 | 0.24084 | 0.252808 | 0 | 0 | 0 |
| GT1 | 18.15676 | 16.29249 | 18.098873 | 20.89295 | 18.01762 | 13.98927 |
| GT10 | 8.04184 | 8.81765 | 7.516995 | 4.02487 | 4.18383 | 6.295109 |
| GT105 | 1.58018 | 1.60096 | 1.189821 | 2.04396 | 1.87823 | 2.17951 |
| GT16 | 3.46227 | 3.54082 | 3.813272 | 3.90922 | 4.87448 | 5.626098 |
| GT2 | 175.52857 | 168.24946 | 148.763578 | 66.58332 | 73.33795 | 82.876319 |
| GT20 | 10.90537 | 9.76982 | 10.090323 | 10.78479 | 15.14046 | 13.405584 |
| GT23 | 5.6811 | 5.5411 | 6.891242 | 6.40862 | 6.48398 | 4.024958 |
| GT24 | 3.95208 | 3.59556 | 3.517071 | 3.26066 | 3.36796 | 4.448778 |
| GT26 | 19.80455 | 19.49467 | 20.24196 | 4.99161 | 4.14847 | 3.615404 |
| GT27 | 16.7568 | 15.60274 | 16.474661 | 17.66331 | 19.46359 | 26.652521 |
| GT3 | 8.53999 | 8.80103 | 6.867327 | 13.42693 | 10.88728 | 9.568459 |
| GT30 | 0.83467 | 0.77535 | 0.699395 | 0.8652 | 0.81027 | 0.512223 |
| GT31 | 4.98589 | 4.31332 | 4.358605 | 4.82532 | 6.56997 | 5.20755 |
| GT32 | 20.76684 | 21.50588 | 22.417573 | 4.4511 | 3.9614 | 3.256708 |
| GT35 | 7.76091 | 8.71728 | 9.052749 | 8.49327 | 8.89671 | 14.269008 |
| GT4 | 40.35549 | 39.48727 | 38.742746 | 9.3313 | 10.23366 | 7.910369 |
| GT41 | 8.73387 | 8.88888 | 8.016147 | 12.38261 | 10.23896 | 11.764694 |
| GT43 | 1.44714 | 1.69302 | 1.591278 | 2.11063 | 2.08563 | 2.801591 |
| GT47 | 16.84757 | 17.67161 | 15.257996 | 18.93098 | 23.6496 | 21.813007 |
| GT5 | 1.63869 | 1.96164 | 1.789542 | 0.13108 | 0.09664 | 0.110005 |
| GT51 | 14.31027 | 12.63446 | 12.738691 | 3.5212 | 4.27634 | 5.372313 |
| GT61 | 1.7927 | 1.78309 | 1.762953 | 3.27261 | 2.53647 | 3.41406 |
| GT62 | 0.34399 | 0.39103 | 0.346357 | 0.17623 | 0.14463 | 0.155933 |
| GT64 | 16.1718 | 17.72447 | 15.880906 | 18.51981 | 24.69973 | 21.174047 |
| GT66 | 18.23016 | 19.22164 | 16.047524 | 17.87861 | 18.63818 | 10.386383 |
| GT7 | 6.78396 | 7.23719 | 6.469294 | 12.09573 | 9.38554 | 10.579094 |
| GT8 | 328.38935 | 340.89895 | 280.346055 | 500.54299 | 405.08852 | 366.741624 |
| GT90 | 1.77695 | 1.74353 | 2.243534 | 1.59314 | 1.46155 | 1.558224 |
| GT98 | 3.11134 | 3.37996 | 3.251692 | 3.90255 | 4.51885 | 4.084685 |
| PL0 | 15.34959 | 14.68709 | 15.439168 | 3.56461 | 4.37291 | 3.940044 |
| PL14 | 0.701 | 0.79373 | 0.70385 | 0.39064 | 0.31996 | 0.347236 |
| PL3 | 12.52734 | 12.01493 | 12.192202 | 12.41476 | 17.24888 | 15.640236 |
| PL4 | 0.49901 | 0.49469 | 0.483638 | 5.33949 | 5.99676 | 7.795356 |
| PL8 | 12.26704 | 13.11733 | 15.328183 | 2.31871 | 3.09763 | 2.955502 |

**Supplementary Table 6** Selection of bacterial genes involved in biodegradation of carbohydrates

| Function | KO ID | Gene name | Relative Abundance | | Known activity |
| --- | --- | --- | --- | --- | --- |
|  |  |  | Rice | Maize |  |
| lignin biodegradation | K03781 | katE, CAT, catB, srpA | 6.988913 | 7.313956 | catalase |
|  | K03782 | katG | 0.946034 | 0.33784 | catalase-peroxidase |
|  | K00432 | gpx | 33.64295 | 14.71044 | glutathione peroxidase |
| Cellulose and hemicellulose biodegradation | K00694 | bcsA | 13.38927 | 3.15672 | cellulose synthase (UDP-forming) |
|  | K05349 | bglX | 83.71271 | 17.79225 | beta-glucosidase |
|  | K05350 | bglB | 72.57746 | 35.02341 | beta-glucosidase |
|  | K07406 | melA | 56.355493 | 11.439267 | alpha-galactosidase |
|  | K07407 | E3.2.1.22B, galA, rafA | 41.549269 | 8.888532 | alpha-galactosidase |
|  | K01206 | FUCA | 76.165743 | 24.045741 | alpha-L-fucosidase |
|  | K01191 | MAN2C1 | 54.339983 | 14.686688 | alpha-mannosidase |
|  | K01209 | abfA | 73.30846 | 17.634609 | alpha-L-arabinofuranosidase |
|  | K01224 | E3.2.1.89 | 14.445472 | 2.685678 | arabinogalactan endo-1,4-beta-galactosidase |
|  | K01190 | lacZ | 50.195314 | 10.330846 | beta-galactosidase |
|  | K01195 | uidA, GUSB | 17.650803 | 14.109157 | beta-glucuronidase |
|  | K01192 | E3.2.1.25, MANBA, manB | 36.891656 | 21.725957 | beta-mannosidase |
|  | K03928 | yvaK | 19.275126 | 3.179536 | carboxylesterase |
|  | K01179 | E3.2.1.4 | 29.175414 | 6.218657 | endoglucanase |
|  | K01218 | gmuG | 16.655519 | 3.360171 | mannan endo-1,4-beta-mannosidase |
|  | K01222 | E3.2.1.86A, celF | 42.832675 | 9.185885 | 6-phospho-beta-glucosidase |
|  | K01188 | E3.2.1.21 | 7.859204 | 7.878041 | beta-glucosidase |
| Xylan biodegradation | K01181 | E3.2.1.8, xynA | 15.915317 | 3.113641 | endo-1,4-beta-xylanase |
|  | K01198 | xynB | 43.396382 | 9.131097 | xylan 1,4-beta-xylosidase |
| Pectin | K01051 | E3.1.1.11 | 35.025787 | 8.400352 | pectinesterase |

**Supplementary Table 7** KEGG pathways analyses of the three most abundant *Enterococcus* species, *E.* sp. FDAARGOS-375, *E. casseliflavus* and *E. gallinarum*

|  |  |
| --- | --- |
| Central carbohydrate metabolism | |
| M00001 | Glycolysis (Embden-Meyerhof pathway), glucose => pyruvate (complete 9/9) |
| M00002 | Glycolysis, core module involving three-carbon compounds (complete 5/5) |
| M00003 | Gluconeogenesis, oxaloacetate => fructose-6P  (complete 7/7) |
| M00307 | Pyruvate oxidation, pyruvate => acetyl-CoA  (complete 1/1) |
| M00010 | Citrate cycle, first carbon oxidation, oxaloacetate => 2-oxoglutarate  (complete 3/3) |
| M00006 | Pentose phosphate pathway, oxidative phase, glucose 6P => ribulose 5P  (complete 2/2) |
| Other carbohydrate metabolism | |
| M00631 | D-Galacturonate degradation (bacteria), D-galacturonate => pyruvate + D-glyceraldehyde 3P  (complete 5/5) |
| M00061 | D-Glucuronate degradation, D-glucuronate => pyruvate + D-glyceraldehyde 3P (complete 5/5) |
| M00632 | Galactose degradation, Leloir pathway, galactose => alpha-D-glucose-1P (complete 4/4) |
| M00854 | Glycogen biosynthesis, glucose-1P => glycogen/starch   (complete 2/2) |
| M00549 | Nucleotide sugar biosynthesis, glucose => UDP-glucose (complete 3/3) |
| M00554 | Nucleotide sugar biosynthesis, galactose => UDP-galactose  (complete 2/2) |
| M00909 | UDP-N-acetyl-D-glucosamine biosynthesis, prokaryotes, glucose => UDP-GlcNAc  (complete 5/5) |
| Amino acid metabolism | |
| M00018 | Threonine biosynthesis, aspartate => homoserine => threonine (complete 5/5) |
| M00021 | Cysteine biosynthesis, serine => cysteine  (complete 2/2) |
| M00017 | Methionine biosynthesis, apartate => homoserine => methionine  (complete 7/7) |
| M00019 | Valine/isoleucine biosynthesis, pyruvate => valine / 2-oxobutanoate => isoleucine  (complete 4/4) |
| M00570 | Isoleucine biosynthesis, threonine => 2-oxobutanoate => isoleucine  (complete 5/5) |
| M00432 | Leucine biosynthesis, 2-oxoisovalerate => 2-oxoisocaproate  (complete 3/3) |
| M00028 | Ornithine biosynthesis, glutamate => ornithine  (complete 4/4) |
| M00844 | Arginine biosynthesis, ornithine => arginine  (complete 3/3) |
| M00015 | Proline biosynthesis, glutamate => proline  (complete 2/2) |
| M00026 | Histidine biosynthesis, PRPP => histidine  (complete 6/6) |
| M00023 | Tryptophan biosynthesis, chorismate => tryptophan  (complete 3/3) |
| Metabolism of cofactors and vitamins | |
| M00125 | Riboflavin biosynthesis, plants and bacteria, GTP => riboflavin/FMN/FAD  (complete 7/7) |
| M00120 | Coenzyme A biosynthesis, pantothenate => CoA  (complete 3/3) |
| M00140 | C1-unit interconversion, prokaryotes  (complete 3/3) |
| M00116 | Menaquinone biosynthesis, chorismate (+ polyprenyl-PP) => menaquinol  (complete 9/9) |
